# The Prevalence and Compliance of Health Claims on Food Supplements with Regulation (EC) No. 1924/2006 Sold In-Store and Online Within the Republic of Ireland

**DOI:** 10.3390/foods15020286

**Published:** 2026-01-13

**Authors:** Nicole Barrow, Leane Hoey, Hans Verhagen

**Affiliations:** 1 School of Biomedical Sciences, Faculty of Life and Health Sciences, Ulster University, Coleraine BT52 1SA, UK; barrow-n2@ulster.ac.uk (N.B.); l.hoey@ulster.ac.uk (L.H.); 2 National Food Institute, Technical University of Denmark, Kemitorvet 201, 2800 Kongens Lyngby, Denmark; 3 Food Safety & Nutrition Consultancy, Couwenhoven 4214, 3703 EE Zeist, The Netherlands

**Keywords:** food supplements, health claims, EU Regulation 1924/2006, EU Regulation 432/2012, EU Register of Health Claims

## Abstract

The food supplement market has expanded rapidly in recent years, driven by demand for health, wellness, and healthy ageing; yet, the integrity of associated Health Claims (HC) remains uncertain. In the European Union (EU), food supplements are regulated under Directive 2002/46/EC, while HC use is governed by Regulation (EC) No. 1924/2006 (NHCR), which requires scientific substantiation evaluated by the European Food Safety Authority and subsequent authorisation by the European Commission/Member States. Despite this framework, concerns persist regarding unauthorised or non-compliant HC. This study examined the prevalence and compliance of HC on food supplement labels in the Republic of Ireland, comparing products sold in-store and online. A total of 192 food supplements were randomly selected across multiple categories, with HC compliance assessed against the EU Register of Nutrition and Health Claims and mandatory labelling requirements. In total, 2604 HC were identified, with multivitamins and botanicals as the most common categories reviewed. Although most HC referred to vitamins D and C and focused on immune function, only 80.7% of in-store claims and 75.6% of online claims were authorised, and only around one-third used the prescribed wording. Compliance was notably lower for botanicals, reflecting regulatory ambiguities around their use. These findings highlight persistent challenges in enforcing the NHCR, particularly for online sales and botanicals, and underscore the need for greater regulatory clarity and consumer protection.

## 1. Introduction

Public interest in diet, fitness, and preventative health has driven rapid growth in the global food supplement market, with the EU market projected to grow from $22.4 billion in 2025 to $26.4 billion in 2030. At present, specialty food supplement stores account for 46% of the market share, while online e-commerce of food supplements is expected to grow at a rate of 13% per year between 2024 and 2030 [1]. Food supplements are concentrated forms of nutrients intended to supplement the normal diet, which include vitamins, minerals, amino acids and botanical products. They are marketed in dose form and sold in a variety of formats such as capsules, tablets, powders, liquids, and gummies. According to Food Supplements Europe (FSE), almost nine in ten Europeans consume food supplements, with vitamin D, vitamin C, and magnesium being the most consumed products. Common reasons to use food supplements are to maintain overall health and to support the immune system [2]. In the EU, food supplements are governed under general food law, specifically Directive 2002/46/EC [3]. Given consumer demand for food supplements, the use of Health Claims (HC) in advertisements and on the label can be used as an effective tool to market these products, potentially influencing consumer purchasing behaviour [4].

To prevent misleading consumers, most countries have implemented a regulatory framework to control the promotion of food supplements and their related HC [5]. In the EU, the use of HC on food supplements is regulated by ‘Regulation (EC) No. 1924/2006’ on Nutrition and Health Claims (NHCR). The NHCR appertains to all voluntary claims made on food, supplements, and dietetic products, with an aim to harmonise, regulate, and protect consumers from misleading claims. HC on labels must be truthful, non-misleading, not fear-inducing, and must not encourage excessive or unbalanced eating. Specifically, HC are claims that state, suggest, or imply that there is a direct link between consumption of the food product and health benefits and are divided into functional HC, disease risk reduction claims, and claims referring to children’s development (full details with examples are provided in Appendix A, Table A1). HC require proven benefits, sufficient nutrient levels in consumable form, consumer clarity, and compliance with specific regulatory conditions [6]. The authorisation procedure involves the Panel on Nutrition, Novel Foods and Allergens (NDA Panel) of the European Food Safety Authority (EFSA) assessing the scientific substantiation of the claim and issuing either a positive or negative opinion regarding its suitability. Three key questions are assessed to establish whether a cause-and-effect relationship exists between the food and the claimed effect: (1) is the food on which the claim is made sufficiently defined or characterised? (2) is the claimed effect sufficiently defined and has a beneficial physiological effect? and (3) have pertinent human studies been used to substantiate the claim? [7]. Once EFSA publishes its opinion, the European Commission-approved HC and its wording are added to the EU Register of Nutrition and HC to allow full transparency to the public.

Despite the robust regulatory framework, evidence shows the use of non-authorised or non-compliant HC, suggesting consumer protection is often secondary to the persuasion to buy the product [5]. A study from Ulster University found that HC compliance was high overall, ranging from 90 to 94% in the food categories studied, which included dairy products, fruit-based beverages, and teas, but excluded supplements. Violations across food categories were due to non-compliant wording or the use of non-authorised HC [8]. Conversely, a 2021 study on HC on food supplements made on Spanish Radio, found that 80.3% of functional HC, and 100% of the reduction in disease risk claims made on radio were not authorised by the EU. The claims used were vague or false and omitted essential information. The use of clinical terms such as ‘symptoms’, ‘risk factors’, and ‘dosage’, as if the product was prescribed by a physician, falsely promoted the benefit of the food supplements to the consumer [5]. Similarly, a 2018 study reviewing the quality of information relating to food supplements on the internet, found that all regulatory criteria relating to labelling were only successfully fulfilled by 11.3% of websites studied. In regards to HC, only 46.5% of websites met the regulatory criteria [9]. These studies, conducted in other European, countries suggest there is a high prevalence of non-compliant HC on food products. The presence of unauthorised, false HC can lead to misinformation about the benefits of food supplements to consumers [5].

Botanical food supplements pose distinct challenges for HC compliance. Derived from plants, algae, fungi, or lichens, these products have long been used in Europe, yet concerns remain about their safety and quality [10]. Their use is governed by general EU rules and national laws, with nineteen Member States maintaining their own lists of permitted or prohibited plant substances. Only HC for botanicals are harmonised under the NHCR. When EFSA first evaluated botanical claims in 2009, none received a positive opinion, largely due to a lack of human intervention studies and inconsistent acceptance of “traditional use” evidence. As a result, the authorisation process was suspended in 2012, and an on-hold list of 2078 claims was created [11]. These claims may still be used if operators comply with NHCR principles while awaiting a final decision. The results of The European Commission’s regulatory fitness and performance programme (REFIT) found that consumers are exposed to unsubstantiated claims from this list and may incorrectly assume they have been scientifically verified by EFSA, meaning the NHCR’s objectives are not being fully achieved [12].

Research into the compliance of HC as it pertains to the NHCR is limited, especially for food supplements, thus further research is required in this area. Dublin, Ireland is a member of the EU and therefore implements EU food legislation directly while also maintaining national guidance and enforcement structures, e.g., the Food Safety Authority Ireland (FSAI). This makes it a strong example for how EU Regulations operate in practice. Therefore, the aim of this study was to explore the prevalence and compliance of HC made on food supplement labels on products sold in-store compared to online, in Dublin, Ireland, according to the NHCR.

## 2. Materials and Methods

### 2.1. Study Design

The study design was based on previous research at Ulster University that reviewed the prevalence and compliance of HC on prepackaged foods sold in three major supermarkets in Great Britain (GB). The food categories included dairy products, fruit juices, and teas, and 440 products were surveyed. The researchers physically examined packages on supermarket shelves and compared the information to what was present on the corresponding supermarket online shopping site. For each product the presence, type, and wording of the HC were recorded, which was then cross checked with the GB Register of Nutrition and Health Claims, and wording requirements from Regulation 1924/2006 [8]. In the current study, categories of food supplements were selected at random based on those available in two participating retail stores, a grocery store and a health food store, chosen to represent popular retailers in Dublin, Ireland. Within each selected category, products were chosen consecutively from the shelf (left to right/top to bottom) to avoid subjective selection, until approximately 200 products had been collected. The stores were contacted for permission to conduct the study. Regarding distance selling, Article 14 of Regulation (EU) No. 1169/2011 states that mandatory food information must be present before purchase, except for the date of minimum durability. Information on HC must also comply with this; therefore, the information that is present online must reflect what is sold in-store to the consumer [13]. What was found on labels in-store was compared to the information presented on their online shopping website. No statistical power calculation was performed as this is an under-researched topic; therefore, an exploratory analysis was conducted. Ethical approval was not required.

### 2.2. Data Collection

**Figure 1:**
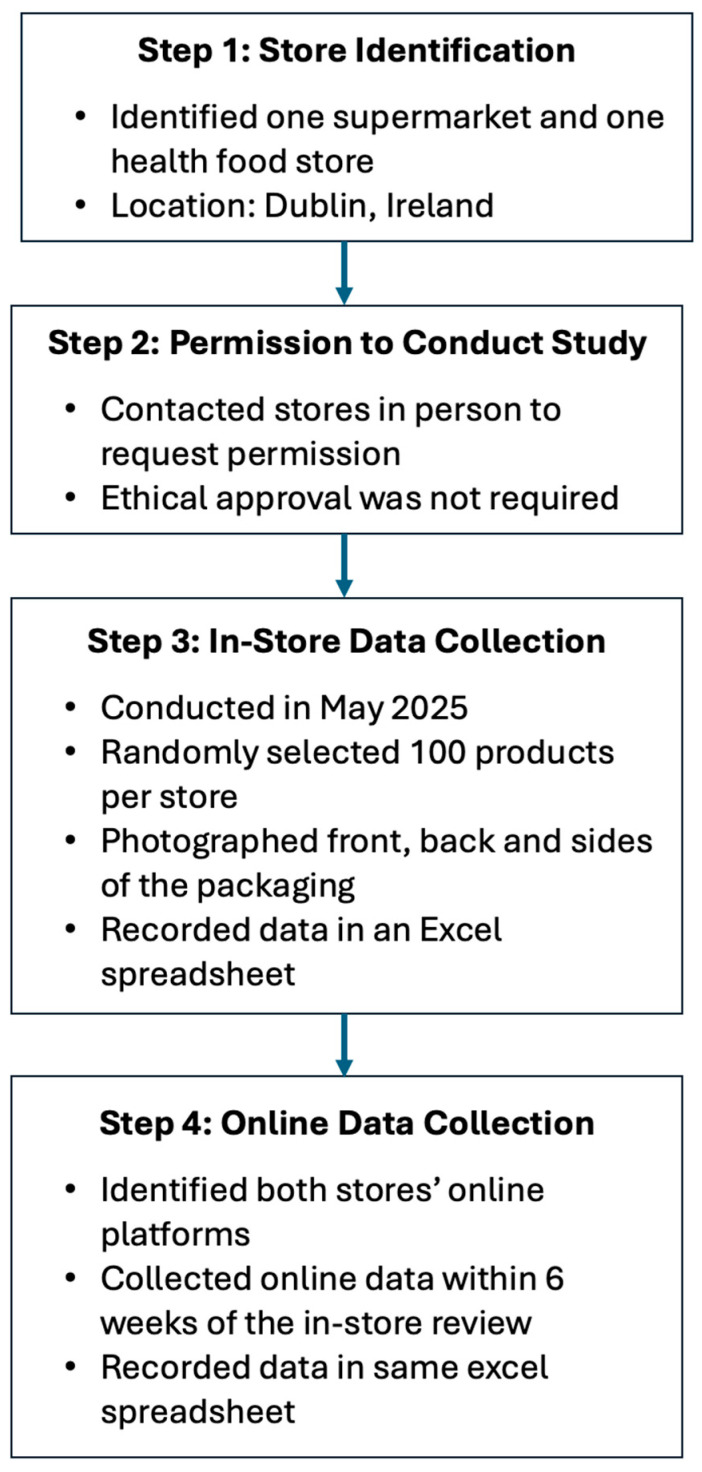
Flow chart summarising the data collection process.

Throughout May 2025, product information was collected on 192 food supplements which were reviewed across two retail outlets, of which 180 were also available on the corresponding e-commerce platform. In-store, food supplements were chosen at random, and pictures were taken of the front, back, and side of each label; the information was subsequently extracted and added to an excel document. The parameters that were extracted included: how it was sold (in-store or online); the product, type and format of food supplement, and target group; if a HC was present (Y/N); number and type of HC present; nutrient(s) which the claim refers to; the HC category (e.g., bone health, immune system, and normal metabolism); if the HC was authorised or not as per EU Register of Nutrition and Health Claims; if the HC was overall compliant (Y/N); if the wording was compliant (Y/N) and if the HC had the exact wording prescribed by Regulation (EU) 432/2012; if mandatory HC information was present (Y/N); if mandatory food supplement information was present (Y/N); and if the claim was present front-of-pack or back-of-pack. There was no overlap on products between the two stores. The data collection process is summarised in Figure 1.

### 2.3. Health Claim Compliance Assessment

**Figure 2:**
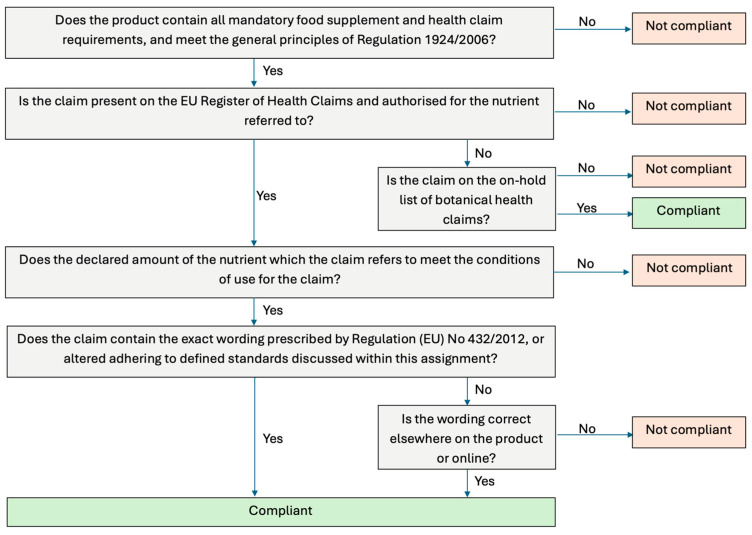
Flow chart summarising the health claim compliance assessment.

To ensure consistency, HC compliance was evaluated using the standardised parameters listed in Figure 2. Mandatory information for food supplements was checked according to Article 6 of Directive 2002/46/EC, requiring: the name of the nutrient, the portion of the product recommended for daily consumption, a warning not to exceed the recommended dose, a statement that the food supplement should not substitute a varied diet, and a statement that products should be stored out of reach of children [3]. Mandatory requirements for HC were assessed under Article 10 of Regulation (EC) No 1924/2006, including: a statement indicating the importance of a varied, balanced diet and healthy lifestyle; quantity of food and pattern of consumption required to obtain the claimed effect; if needed, a statement addressed to the persons who should avoid consuming; and an appropriate warning if the product is likely to present a health risk if excessively consumed [6]. All information relating to mandatory requirements for both food supplements and HC needed to be present, along with meeting the general principles of Regulation 1924/2006 in order for the product to be considered compliant.

The EU Register of Nutrition and HC was then consulted to verify whether each HC was authorised for the nutrient that it referred to and met the conditions of use. For example, ‘vitamin B6 contributes to the reduction of tiredness and fatigue’ can be used ‘only for food which is at least a source of vitamin B6’ [14]. Most of the HC required that the product contain a ‘source of’ the vitamin or mineral. A claim that a food is a source of vitamins or minerals, or any claim likely to convey the same meaning to the consumer, may only be made if the product contains at least a “significant amount”, defined in Directive 90/496/EEC as 15% of the recommended allowance per 100 g, 100 mL, or per package if it contains a single portion [15]. Directive 90/496/EEC applies this threshold to nutrition labelling of foods, not food supplements, which fall under Directive 2002/46/EC; food supplements must declare nutrients per daily recommended portion rather than per 100 g or mL [3]. Regulation (EC) No. 1924/2006 further clarifies that for a claim to be truthful the substance must be present in sufficient amounts to produce the claimed nutritional or physiological effect and be bioavailable. Accordingly, for food supplements, a “significant amount” is 15% of the nutrient reference value (NRV), based on the recommended daily consumption, rather than per 100 g or 100 mL [16].

To assess whether or not the wording of the claim was compliant, the FSAI guidance for the wording of HC was followed: the adapted wording must have the same meaning as the authorised wording and must not be exaggerated or stronger; the word ‘normal’ must not be replaced; when reference is made to a general, non-specific benefit of a nutrient, it must be accompanied by a specific authorised claim; the HC must only be made for the nutrient or food category which have been authorised and not for the product or brand that contains them; and the wording must come from the EU Register of Nutrition and HC [17]. Standardised categories were created to ensure a consistent approach in recording the type of HC (Table 1).

Botanicals went through a similar assessment to the other categories of food supplements to confirm compliance, outlined in Figure 2. An additional step was taken to confirm if the claim was on the on-hold list for botanical HC or if it was on the EU Register of Nutrition and Health Claims.

### 2.4. Statistical Analysis

The data was analysed using SPSS Statistics (Version 29.0). Descriptive statistics were used to provide an initial overview of the dataset, including the number and proportion of products carrying health claims, the frequency of compliant versus non-compliant claims, and how these distributions varied between in-store and online retail settings. Chi-square tests were used to assess differences in compliance rates between food supplement categories across in-store and online settings. This test was applied because the analyses involved comparing categorical outcomes (e.g., authorised vs. not authorised; correct wording vs. incorrect wording; compliant vs. non-compliant) across two independent groups (in-store vs. online). These variables represent counts or frequencies rather than continuous measurements, making the Chi-square test of independence the appropriate statistical method. A *p*-value < 0.05 was considered statistically significant.

## 3. Results

### 3.1. Prevalence of Health Claims on Food Supplement Categories

A total of 192 food supplements were reviewed, 180 of which were available online. Food supplements were most commonly present in tablet format (42%), followed by capsules (22%) and gummies (15%). Adults of both sexes were the primary target group for HC, with 83% of food supplements directed at them, followed by children (10%), and then women specifically (4%). In-store, 67% of the HC appeared on the back-of-pack. At least one HC was present on 89% of in-store products and 93% of online products carried a HC. In-store, 1236 HC were identified (0–39 claims per product), compared to 1368 online (0–36 per product). Overall, the average number of HC on a product in-store was six, compared to online at seven. Multivitamins accounted for one-third of products reviewed, followed by botanicals and single vitamins. Of all food supplement types investigated, essential fatty acids had the highest average number of HC on products in-store at nine HC per product, whereas amino sugars had the highest average of HC for products sold online at twelve HC per product. Although essential fatty acids only comprised approximately 12% of all products reviewed, they accounted for the second-highest proportion of HC at 16.4% in-store and 13.7% online (Table 1).

### 3.2. Types of Health Claims

**Figure 3:**
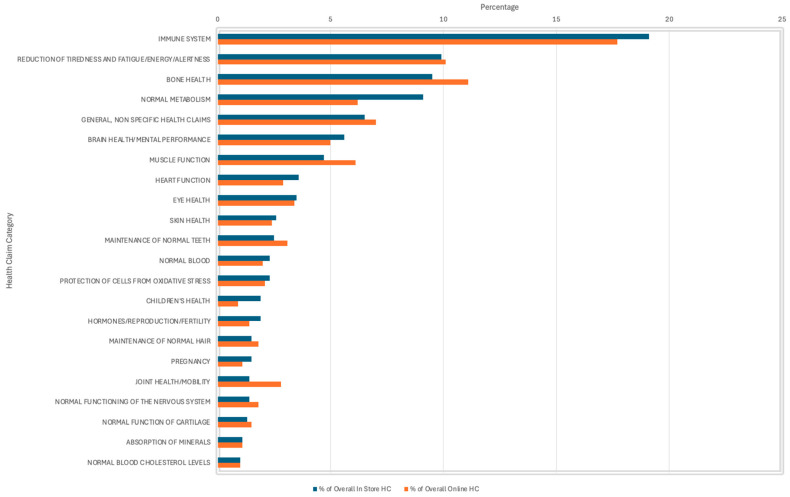
Prevalence of types of Health Claims (HC) on products reviewed in-store and online—all health claim categories are not shown in this analysis.

From the 1236 HC present in-store, 79.1% were Article 13.1 function claims that are supported by generally accepted scientific evidence, which directly link the nutrient to the HC (e.g., vitamin C contributes to the normal function of the immune system) [11]. Similarly, of the 1368 HC present online, 74.4% were Article 13.1 claims. Claims about the immune system were the most frequently used, representing 19.1% in-store and 17.7% online. Claims about the reduction in tiredness and fatigue/energy/alertness were the second most prevalent in-store at 9.9% compared to bone health at 11.1% online. General, non-specific HC accounted for 6.5% of the overall HC in-store and 7% of the overall HC online (Figure 3).

### 3.3. Nutrient Used in Health Claims

**Figure 4:**
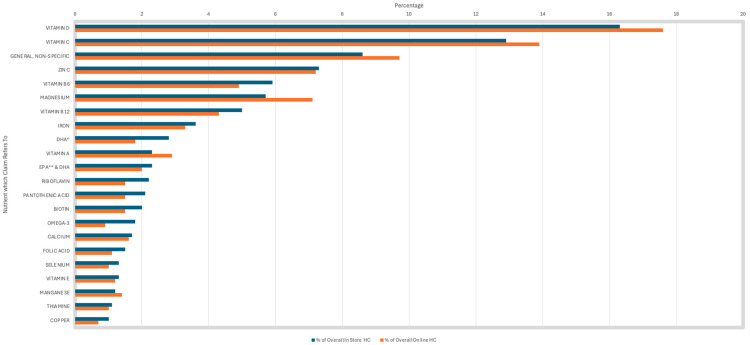
Prevalence of nutrient/constituent to which the health claims refers to—not all nutrients are present in this analysis. * DHA: docosahexaenoic acid. ** EPA: eicosapentaenoic acid.

Vitamin D was the most common nutrient referred to in both in-store and online HC at 16.3% and 17.6%, respectively (Figure 4). Interestingly, vitamin D was most linked to the claims ‘bone health’ and ‘muscle function’, rather than ‘immune system’ claims. Vitamin C was the second most prevalent nutrient on HC sold in-store (12.9%) and online (13.9%). Vitamin C was predominantly linked to immune system claims. General, non-specific nutrients were the third most common constituent used in HC in-store (8.6%) and online (9.7%), and were solely linked to general, non-specific HC.

### 3.4. Health Claim Compliance

Overall, HC compliance was low in-store (58.7%) and even lower online (48.8%). Of the HC present in-store, 80.7% of the products were authorised compared to 75.6% online (Table 2; χ^2^(1) = 10.02, *p* = 0.002), with 35.9% and 30.2% of the HC, respectively, using the exact wording prescribed by Regulation (EU) 432/2012 (Table 3). Compliance among authorised claims was higher in-store (70.1%) than online (62.1%) (χ^2^(1) = 14.82, *p* < 0.001; Table 3). Claims about immune system accounted for 16.3% of the total non-compliant claims made in-store and online, followed closely by general non-specific HC at 14.7% of the total non-compliant claims. Even though reduction in tiredness and fatigue/energy/alertness was the second highest category of HC found on products in-store and third highest online, it only accounted for 4.9% of the total non-compliant HC.

### 3.5. Botanicals

Overall, the majority of HC used on botanical products in-store (48.6%) and online (45.25) were from the EU Register of Nutrition and Health Claims. The category of claims that were most frequently present on botanical products were claims about the immune system. The use of HC from the on-hold list was moderate in-store (28.5%) with higher use rates online (33.1%). Compliance rates were low for HC on botanical products overall, with 59% of HC being non-compliant in-store and 68% of HC being non-compliant online. Specifically, only 17.4% of the authorised HC used in-store were compliant and only 19.1% were compliant online. Of the on-hold HC used, only 16% were compliant in-store and only 8.9% were compliant online (Table 4).

## 4. Discussion

This study contributes to the scarce research on food supplements and their use of HC, specifically within the EU, where the NHCR (Regulation (EC) 1924/2006) governs the authorisation, wording, and use of health claims. This regulatory framework provides the context for assessing compliance in both in-store and online settings in the Republic of Ireland. Prevalence of HC was high, with around 90% of products carrying at least one HC, although overall compliance was low, particularly online. Due to the scarcity of studies specifically examining HC on food supplements, the present findings were interpreted in relation to available literature on HC made for conventional food products and food supplements where possible.

The prevalence of HC on food supplements reviewed in-store and online (89.1% and 92.8%, respectively) was found to be considerably higher than in previous investigations. A study by Lalor et al. found that 17.8% of conventional food products found in-store contained at least one HC. Yoghurt and yoghurt drinks had the highest prevalence of HC at 50%, followed by breakfast cereals at 42% [18]. The data was collected in October 2007, which was one year after the NHCR came into force. Foods that contained HC on their labels prior to the implementation of NHCR were allowed to be used until the products expiry date. As a result, HC that were not authorised or were subsequently rejected by EFSA were allowed during this transition period [19]. In comparison, a 2022 study by Offe et al. on the impact of time on nutrition and HC on the Irish market found that 10.5% of conventional food sold in a supermarket contained at least one HC. Fruit juices and smoothies had the highest rate of HC present with 31.8% of products carrying at least one, while pasta, rice, and bread contained the lowest rates at <1% of products containing a HC. The prevalence of HC on conventional foods was found to have decreased over a period of thirteen years [19]. Similarly, a 2016 study that examined the prevalence of HC on pre-packaged foods across five EU countries (United Kingdom prior to Brexit, the Netherlands, Germany, Slovenia and Spain), found that there were twice as many foods that carried nutrition claims compared to foods that carried HC, 21% and 11%, respectively. The average number of HC per product in-store across the five countries was 1.9 for conventional food [20], which is much lower than the average of 6 HC per food supplement product seen in-store. Interestingly, Hieke et al. also examined the use of symbolic HC, defined as a HC that was ‘pictorial or included both words and pictures’ and found that almost all HC in the Netherlands were symbolic. A limitation of this current study is that symbolic HC were not examined. Taken together, the evidence suggests that while the prevalence of HC on conventional foods has decreased since the implementation of the NHCR, food supplement products across EU markets continue to carry them at disproportionately high prevalence, reflecting both the high consumer demand and regulatory challenges in monitoring these products.

The most commonly used claims referred to the immune system and were predominantly in relation to vitamin C. A recent study by Arora et al. noted the surge in supplement use and immunity related claims in a post COVID-19 environment. Vitamin C was highlighted as the nutrient most often recognised with immune supporting effects [21]. Flu-like sickness or common colds are the most common reported cause of absence from work amongst adults. A recent study by Decke and Seifert found that 82% of all vitamin C supplemental products in Germany and 98% of products in the United States rely on the claim that vitamin C boosted the immune system function and contributed to the normal function of the immune system [22]. Coates et al. found similar results, with immunity claims being the most prevalent in conventional food products at 26.8% in-store and 26.6% online [8]. These patterns suggest that immunity-related claims, especially those centred on vitamin C, continue to dominate both food supplement and conventional food sectors, a trend further reinforced in the post-COVID marketplace.

The compliance of HC in accordance with the NHCR was investigated in this study and it was found that significantly less HC were both authorised and compliant online compared to in-store. These results are consistent with the findings by the French Consumer Protection Authority, who audited 75 food supplement e-commerce platforms and found that 44% of food supplements online had unauthorised HC; 51% used therapeutic claims and 40% were missing mandatory information that must be included on point of sale [23]. To compare this to in-store levels of compliance, a recent study conducted in Serbia, a country that is not in the EU but follows EU regulations, found that on omega-3 supplements 13% of products carried unauthorised HC [24]. Collectively, these results reinforce the difficulty of ensuring NHCR-compliant communication in digital retail environments, suggesting that online markets continue to present a greater regulatory challenge than traditional in-store settings. This is corroborated by a 2018 report from the European Commission which concluded that controls over food sold online were limited and focused on the registration of the food business operator. Most non-compliances reported were related to labelling and improper use of health claims. The report notes that the food business operator has the ability to rapidly enter or exit the online marketplace, making it difficult to patrol with limited resources [25]. To address the persistent compliance gaps in online food supplement sales, EFSA and national authorities such as the FSAI could strengthen enforcement through targeted monitoring, increased platform accountability, guidance documents for digital labelling, and better allocation of inspection resources, ultimately improving consumer protection and adherence to the NHCR.

It was observed that both in-store and online, approximately 30% of authorised HC used the exact wording prescribed by Regulation (EU) 432/2012. This suggests that the wording is not understandable by consumers. In a European Institute of Innovation and Technology (EIT) Food-funded study, ‘health claims unpacked’, it was explored how the wording of a HC is understood by consumers of different backgrounds. The study showed that consumers find HC difficult to understand, and consumers do not trust that they are accurate. The research also concluded that there is a need for new guidelines on wording to consider language and cultural differences between Member States [26]. It has also been shown that a consumer’s understanding of a claim directly relates to the personal relevance of the described benefit [27]. For this reason, claims should be modified for their target group to increase understanding. However, the current EU legislation requires claims to be presented in scientific language, this is a challenge for food manufacturers to make the claim non-misleading, scientifically substantiated, compliant with regulatory wording but also understandable by the consumer [28]. General, non-specific HC, that were not accompanied by a specific HC such as ‘contributes to overall vitality and wellbeing’ and ‘supports your whole-body health’ accounted for 7% of the overall HC both in-store and online, all of which were non-compliant. The NHCR states that “reference to general, non-specific benefits of the nutrient or food for overall good health or health-related well-being may only be made if accompanied by a specific HC included in the lists provided for in Article 13 or 14” [6]. These vague claims could be misleading or not correctly understood by the average consumer [29]. These results are in line with a previous study on the prevalence of HC on prepackaged foods in Europe, which showed that 6.7% of all HC were general, and non-specific [20]. The observed differences in compliance underscore potential risks to consumer trust and safety, reinforcing the importance of comprehensive regulation and transparent product information across all platforms. These comparative findings demonstrate persistent gaps in HC compliance across online and in-store contexts, highlighting the continuing need for increased regulatory oversight.

In this study, botanical food supplements accounted for 12% of the overall HC both in-store and online, with a higher proportion of claims coming from the EU Register of Nutrition and Health Claims, compared to the on-hold list. The compliance rates from the on-hold list of botanical HCs are low, with only 16% being compliant in-store and only 8.9% compliant online. It is important to note that compliance of the on-hold claims was evaluated considering their current status as approved for use, even though they have yet to be assessed by EFSA. When these claims undergo evaluation, their compliance status could become questionable. A recent study by Vojvodic et al., conducted in Serbia, found that 38.7% of supplements reviewed in-store carried compliant on-hold claims from the EFSA Register of questions for botanicals [4]. Comparatively, this number is considerably higher than the 16% compliant on-hold claims found in this current study, this could be due to the fact that Serbian law requires a pre-market registration of food supplements. The result of the current study shows a lack of adherence to Regulation (EC) 1924/2006. This is consistent with the results of the REFIT evaluation, which were published in 2020, and demonstrated that consumers are subject to unsubstantiated HC on botanical products from the on-hold list. Consumers are exposed to potentially misleading information as they may believe that the beneficial effects of the supplements have been scientifically assessed by EFSA [30]. Overall, the low compliance of botanical supplements, especially those on the on-hold list, seems to result from both unclear rules and poor adherence by the industry. Even though EFSA has not yet fully assessed these claims, many are still being used online and in-store, which can mislead consumers into thinking the benefits are scientifically proven. This highlights the need for clearer guidance and better enforcement to make sure health claims on botanical supplements are accurate and trustworthy.

This study provides notable strengths, firstly, it shows a systematic comparison of HC both in-store and on online e-commerce platforms, an approach that remains relatively underexamined even with the rapid growth of e-commerce sites. By reviewing 192 products and examining more than 2600 HC present in-store and online, the analysis offers a robust dataset that captures the scale at which consumers are exposed to these HC. A further strength is that the detailed categorisation of results offer comparison by product type, claim type, nutrient and regulatory compliance.

Nevertheless, the study has limitations that must be acknowledged. Its cross-sectional design provides focused analysis of the market at a single point in time and does not allow for seasonal variation or trends over time. The study was also geographically restricted to the Republic of Ireland, which limits the findings to not include other EU Member States with potentially different enforcement practices. The focus was on HC compliance on labels and online, which did not include symbolic HC. Products were randomly selected and may not be representative of the broader food supplement market. Finally, the focus on labelling and website information, without the assessment of alternative marketing materials, limits the ability to evaluate the true impact of HC on purchasing behaviours and consumer impact. These limitations outline the direction of future research.

## 5. Conclusions

In conclusion, this study provides new exploratory insights into the prevalence and compliance of HC on food supplements labels in the Republic of Ireland, contributing to the limited literature available on this subject within the EU. By systematically comparing products sold in-store and online, the analysis reveals a high overall prevalence of HC, with the majority of food supplements carrying at least one claim. However, compliance with the NHCR was inadequate, particularly for products sold online, where unauthorised or inaccurately worded claims were more common. These findings are consistent with concerns raised in other European contexts, suggesting that despite a robust legal framework, enforcement challenges remain. Of particular concern are botanicals, which represented one of the largest food supplements categories and displayed the lowest levels of compliance. As a result of low levels of compliance of HC from the on-hold list, consumers may be exposed to potentially misleading information, believing such claims to be scientifically validated. This undermines the objectives of the NHCR, which was designed to ensure consumer protection. The study also highlights the difficulties arising from the wording requirements of authorised HC, which may not always be easily understood by consumers. This creates tension between scientific precision and consumer comprehension, leading to a widespread use of adapted or non-compliant phrasing. Given the exploratory nature of this study and its national context, further research is warranted to assess the extent of these issues across a broader range of products, jurisdictions, and regulatory environments. Overall, the findings underscore the need for improved regulatory clarity, especially for botanicals, and enhanced enforcement mechanisms that extend effectively into digital markets. Greater emphasis on consumer understanding of HC, alongside robust compliance monitoring, will be crucial in safeguarding public trust and ensuring food supplements contribute meaningfully to public health.

## Figures and Tables

**Table 1:** Summary of the types of food supplements sold in-store and online, and the prevalence and compliance of Health Claims (HC).

	In-Store					Online					
Category of Food Supplement	Number of Products Reviewed (*n* = 192)	Total Number of HC (*n* = 1236)	% of Overall HCs Present	% Overall Compliance	Average HC per Product	of Which Available Online (*n* = 180)	Total Number of HC (*n* = 1368)	% of Overall HC	% Overall Compliance	Average HC per Product	Most Prevalent Health Claim Category per FS Category
Multivitamins *	62	508	41.1	58.1	8	56	509	37.2	52.1	8	Immune System
Essential Fatty Acids	23	203	16.4	64	9	19	187	13.7	34.2	9	Brain health/mental performance
Botanicals	34	144	11.7	33.3	4	34	157	11.5	28	4	Immune System
Vitamins	31	144	11.7	58.3	5	28	175	12.8	60.6	6	Immune System
Vitamin and Mineral	14	114	9.2	74.6	8	14	125	9.1	78.4	4	Immune System
Minerals	13	72	5.8	79.2	5	13	117	8.6	41.9	8	Reduction in Tiredness and Fatigue/Energy/Alertness
Amino Sugar	4	17	1.4	70.6	4	4	46	3.4	52.2	12	Maintenance of normal bones
Protein	2	16	1.3	50	8	2	17	1.2	0	9	Maintenance of normal bones
Amino Acid	2	8	0.6	62.5	4	2	9	0.7	82.4	4	Normal formation of connective tissue
Fibre	2	4	0.3	50	2	2	9	0.7	66.7	5	Weight management
Plant Sterols	1	2	0.2	0	2	1	4	0.3	75	4	Normal blood cholesterol levels
Probiotic	2	2	0.2	0	1	2	5	0.4	0	3	Digestive health
Other Substances	1	1	0.1	0	1	2	5	0.4	0	2	Joint health/mobility

* May contain minerals.

**Table 2:** Authorisation of Health Claims (HC) on food supplements in-store and online.

	In-Store		Online		
Is the HC Authorised or Not?	Total Number of HC (*n* = 1236 *)	% of Overall HC	Total Number of HC (*n* = 1368 *)	% of Overall HC	*p*-Value
Authorised	997	80.7	1034	75.6	0.002
Not Authorised	160	12.9	236	17.3	0.002

*p*-value obtained from Chi-Square. Statistically significant difference = *p* < 0.05. * HC made on-hold botanical products in-store (*n* = 79) and online (*n* = 98) were not included in this analysis as they do not fall under ‘authorised’ or ‘not authorised’. χ^2^(1) = 10.02.

**Table 3:** Use of exact authorised wording and overall compliance with Regulation (EU) 432/2012 for Health Claims (HC) on food supplements in-store and online.

	In Store		Online		
Exact Authorised Wording Used	Total Number of HC (*n* = 997)	% of Overall HC	Total Number of HC (*n* = 1034)	% of Overall HC	
Yes	358	35.9	312	30.2	
No	632	63.4	712	68.9	
**Overall Compliance of the Authorised HC**					*p*-Value
Compliant	699	70.1	642	62.1	<0.001
Non-compliant	298	29.9	392	37.9	<0.001

*p*-value obtained from Chi-Square. Statistically significant difference = *p* < 0.05. χ^2^(1) = 14.82.

**Table 4:** Authorisation of Health Claims (HC) on botanical food supplements in-store and online and their rate of compliance.

	In Store			Online		
Is the Botanical HC Authorised?	Total Number of HC (*n* = 144)	% of Overall HC	% Compliant	Total Number of HC (*n* = 157)	% of Overall HC	% Compliant
Authorised	70	48.6	17.4	71	45.2	19.1
Not Authorised	22	15.3	0	27	27	0
On-hold List	41	28.5	16	52	33.1	8.9

## Data Availability

The original contributions presented in the study are included in the article, further inquiries can be directed to the corresponding author.

## Abbreviations

The following abbreviations are used in this manuscript:

HC	Health Claim/Health Claims
EU	European Union
EFSA	European Food Safety Authority
NHCR	Nutrition Health Claim Regulation
FSE	Food Supplements Europe

## Appendix A

**Table A1:** Classification and conditions of use of health claims according to Regulation 1924/2006 [6].

Type of Claim	Scope	Example Claim	Conditions of Use of the Claim
**Nutrition Claim (Content and Comparison Claims)**	Refers to statements that suggest or imply that a food possesses particular beneficial nutritional properties due to the presence, absence, increase, or decrease in a nutrient.	“Low fat”	Product must contain <3 g fat/100 g solids or <1.5 g fat/100 mL liquids.
**Health Claim (Article 10.3)**	General, non-specific health claims.	“Good for your skin”	Must be accompanied by an Article 13 or 14 claim.
**Health Claim (Article 13.1)**	Relates to the physiological effect of a nutrient or substance. based on generally accepted scientific evidence.	“Calcium and vitamin D are needed for the maintenance of normal bones”	Food must contain ≥15% of the nutrient reference value (NRV) per 100 g of the product to qualify as a “source.”
**Health Claim (Article 13.5)**	Refers to the physiological effect of a nutrient or substance. based on newly developed scientific evidence.	“Sugar beet fibre contributes to an increase in faecal bulk”	At least 6 g fibre/100 g or 3 g fibre/100 kcal, enabling claim of “high fibre.”
**Reduction in Disease Risk Claim (Article 14.1.a)**	Any statement suggesting that consumption of a nutrient or substance reduces a risk factor for disease development, based on generally accepted scientific evidence.	“Plant sterols and stanol esters have been shown to lower/reduce blood cholesterol. High cholesterol is a risk factor in the development of coronary heart disease.”	Consumers must ingest 1.5–3 g of plant sterols daily to achieve effect. Permitted only for specific foods (e.g., yellow fat spreads, dairy products, mayonnaise, salad dressings).
**Health Claim Referring to Children’s Development (Article 14.1.b)**	Claims suggesting that consumption of a nutrient contributes to normal growth, development, or health of children, based on generally accepted scientific evidence.	“Calcium is needed for normal growth and development of bone in children”	Food must contain ≥15% NRV per 100 g, qualifying as a “source.”
